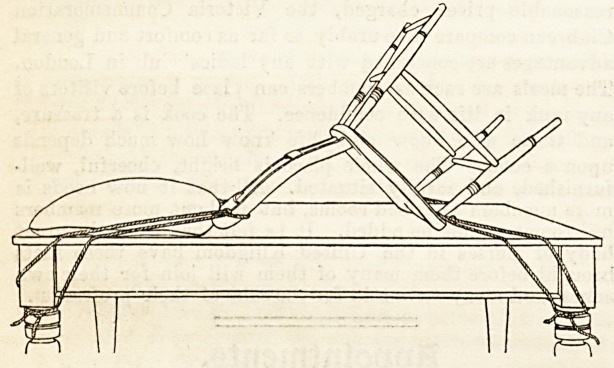# "The Hospital" Nursing Mirror

**Published:** 1897-10-16

**Authors:** 


### "The Hospital" Nursing Mirror.

Being the Nursing Section of "The Hospital."

[Contributions for this Section of "The Hospital" should be addressed to the Editor, The Hospital, 28 & 29, Southampton Street, Strand, London, W.C., and should have the word "Nursing" plainly written in left-hand top corner of the envelope.]

### News from the Nursing World.

### NURSES AT THE EASTERN FEVER HOSPITAL.

The health of the nursing staff at the Eastern Fever Hospital is excellent. The nurses have taken to riding bicycles, and enjoy it very much. A light, pleasant room has been turned into a bicycle stable, and the riders, wrapped in the comfortable woollen black and white checked shawls provided for their use by the hospital authorities, spend many a merry hour in "grooming" their respective steeds. The library for their use also seems admirably managed. About two years ago Mudie kindly sent down a batch of books, and a charge of one penny per volume and a halfpenny for every week it is kept accumulates for the purchase of new books. This plan is popular, as it enables the nurses to obtain current literature, and keeps them in touch with the reading world. The new block built about two years ago provides delightful sitting and dining rooms. They are large, handsome, airy, and cheerful, and the remnants of the midday dinner just about to be cleared away at the time of our visit showed a bill of fare consisting of fish, roast beef, potatoes boiled and roast, and what sailors call "plum duff." The sleeping accommodation, however, is becoming somewhat straitened, for though the greater number of the staff have separate rooms, some of the junior are not so fortunate. The splendid health of the nursing staff in this isolated colony of scarlet fever, diphtheria, and typhoid cases is most encouraging. For what can be done within the wards of a fever hospital can surely be accomplished outside when the general public takes the same sanitary precautions. It is interesting to note that only about 21 cases of typhoid are taken here, it being customary now for the authorities to board out typhoid patients at the general hospitals.

### THE NURSES AT MAIDSTONE.

The Nurses' Co-operation, 8, New Cavendish Street, has already supplied over ninety nurses to Maidstone. Some are working in the temporary hospitals, and many are engaged in district nursing. It is pleasant to hear of the hearty way in which the latter have adapted themselves to a branch of nursing which is new to them. They appear to overlook the personal discomforts and fatigue, inseparable from this house-to-house work, and are one and all well, happy, and useful. They are well looked after, many in private houses, where they meet with the warmest welcome. A "Nurses' Store" has been opened at the Temperance Hotel, Maidstone, and has been stocked by generous friends with every kind of nursing requisite. Without this store to fall back on, the work amongst the sick would have been terribly hampered. As it is, the demands made on the resources of the Nurses' Store by the workers are met promptly by Miss Maule and her assistants. Doctors and nurses concerned with the care of such numbers of typhoid patients can realise the absolute need of a constant supply of nursing requisites. The Princess Christian showed her sympathy in a very practical manner, by sending three trained nurses to the town from Windsor.

### OUR NEEDLEWORK COMPETITION.

Once more we invite contributions to our needlework competition, which will, as usual, be distributed amongst the sick in the hospitals. Every contributor should send name and address in the parcels, and address them, "The Needlework Competition, care of the Editor of The Hospital, 28 and 29, Southampton Street, Strand, London, W.C." They should reach us on December 16th and 17th. Private nurses have special opportunities for co-operating in this excellent work, as those who have leisure to sew in the sick-room are able to give their services, and many patients are to be found willing to contribute materials. We learn from matrons that warm vests are much appreciated and needed for men, women, and children when they leave the shelter of the hospital in cold weather. The nice supply of warm socks we have always received have rejoiced the hearts of many poor patients.

### WANTED—A NURSES' HOME.

The philanthropist in need of an outlet for his purse and energies cannot do better than call at the Chelsea Hospital for Women and master the difficulties for housing the nursing staff. Let him ask the most searching question and pry "upstairs, downstairs, and in my ladies' chamber," and he will thoroughly satisfy himself that the utmost is made of every corner, and that all the resources of the hospital are heavily taxed to accommodate its sixteen nurses and its due number of women domestics. The matron and her charge nurses each have a tiny sleeping apartment, but the rest have to be content with two and even three in the room, whilst the servants' room is very like a ward. In addition the upper half of an old house opposite has been taken and furnished as bed-rooms, and here also two or three nurses are allotted to a room, according to its size. The greatest difficulty to contend with is the want of money. The need of the new home is the more urgent as there is considerable difficulty in obtaining nurses and probationers in a hospital devoted to special diseases, and candidates nowadays generally ascertain if they will have a separate bed-room before engaging themselves.

### RIPON DIAMOND NURSING INSTITUTION.

Hearty co-operation and common-sense have presided over the efforts of the inhabitants of Ripon to commemorate this year's national rejoicing. Once more the cause of the sick has found favour, and the permanent committee of the Ripon Diamond Nursing Institution were unanimously appointed at a meeting in the Town Hall. The nucleus of the funds of the institution is a bequest of Lady Mary Vyner for gratuitously nursing the sick poor, which has hitherto been administered by the wives of the clergy of the Established Church. This fund is now vested in trustees for the use of the new institution, which is non-sectarian. Whilst the wishes of the testatrix will be scrupulously regarded as to the money derived from the fund, the gifts, or part of the gifts, of other subscribers will be used to provide nurses for such as are prepared to pay full or part fees, according to their circumstances, and thus supply the needs of all the residents of the district. One point in the arrangement is likely to promote the successful working of the institution—the qualified medical men practising in Ripon are elected *ex-officio* members of the committee.

### WOMEN'S CONFERENCE AT CROYDON.

THE wife of the Bishop of London, Mrs. Creighton, will preside at the annual conference of the National Union of Women Workers, to be held at Croydon from October 26th to 29th. The subjects to be discussed are many and wide-reaching, and the names of the ladies who will read papers are more or less well-known. Amongst the papers on nursing, one by the well-known authoress of several handbooks on nursing, Miss Honnor Morten, will surely be most instructive; it is entitled, "The Nursing of the Insane and Epileptic."

### QUEEN'S NURSING ASSOCIATION, DARLINGTON.

A VERY pleasant surprise was prepared for the secretary and committee of the Darlington Nursing Association the other day. Mrs. Thorpe Allen inaugurated a sale of work in aid of the Association's fund; and when all was ready, Mrs. A. F. Pease, the lady who has been president of the Association since its birth six years ago, was asked to open it. The sale was held in the sitting-room of the home, and consisted of useful and fancy articles of needlework and flowers. The nurses' work is much appreciated in the town. Accidents, grave illnesses, and convalescents all fall to their care, and their patients take the greatest interest in the Association.

### THE SAMARITAN FUND, HULL.

THOSE most useful adjuncts to our hospitals, the Samaritan Funds, are not infrequently overlooked by the charitable public. Nurses who grow attached to their patients dread for them the day when they must be sent away from the genial warmth and plenty of the ward to the poverty that awaits only too many. It is not, therefore, astonishing to hear that the Matron and nurses of the Royal Infirmary, Hull, bear nearly the whole brunt of maintaining the fund connected with the infirmary. For months past they have spent their leisure in preparing countless dainty trifles as well as useful garments, with the result that a well-patronised sale of work was opened by Mrs. Arthur Wilson in the Ethering Ward. We hope that it will have the effect of awakening wider sympathy for this work amongst the outside public, as it is not at all fair that the hard-working horse should drag the whole cart.

### CROYDON NURSING ASSOCIATION.

THREE nurses on the private nursing staff of the Croydon Nursing Association have been so unfortunate as to become ill in discharge of their duties during the past year. One took fever from her patient, and two injured themselves lifting invalids. In consequence of these misadventures there was no balance to hand on the district branch of the work. This year has not been, therefore, so satisfactory as the past. Mr. Frederick Edridge, explaining this, said: "They relied upon the private nursing to supplement the district nursing account," and "that their principal object in carrying on the institution was for the purpose of securing district nursing." The principle of exploiting the earnings of a private nursing staff on behalf of district nursing is pernicious. There is nothing noble in supporting any charity out of the pockets of others, and if a district provides nurses for the sick poor it should pay them by subscribing the necessary money. We have no sympathy with the method in vogue at Croydon, and in some other places also. True philanthropy aims at securing the worker her full meed of reward to enable her to provide for her necessities now and her need hereafter. Is it no advantage to a neighbourhood to have a body of trained women in their midst at call when sickness lays them low that they must needs tax the nurse to save their own pockets? This view has probably not occurred to the organisers of the Nursing Institution at Croydon, and possibly if it were strongly represented to them by public opinion they would mend their ways.

### THE JOHNS HOPKINS NURSES.

RADICAL changes have been made in the nursing of the Johns Hopkins Hospital, Baltimore. The greatest is undoubtedly that which secures a three-year course of training to the nurses, "in order," to quote the report, "to provide women better equipped by knowledge and experience for nursing, to secure a more efficient service to the hospital by nurses in the third year, and to obviate the breakdown of health of nurses by attempting to crowd the work of training into two short years." The pupil nurses will not receive payment beyond uniform, text books, and sufficient money for small incidental expenses. It is expected that this rule will elevate the standard of the school, and be in the best interests of the pupils. The limit of work hours, both in the wards and lecture-room, is fixed at ten hours a day, in order to enable the probationer to keep her mind alert and fresh. These regulations, if universal, would of course practically debar women without money from entering the profession, and have put an end at once and for ever as far as this institution is concerned to the plebeian probationer. Some compensation is offered by awards of four scholarships of £40 per annum to the pupil nurse for the first two years and £50 for the last. They are given without respect to the pecuniary circumstances of the successful candidate, but when she is not in need of money she is expected to waive her claim for an impecunious but desirable candidate. Democratic America is indeed fast marching in the byeways of aristocracy, and if deeds must be judged by results, then she is justified in this case, "for the school has never been more prosperous nor efficient than at present."

### SHORT ITEMS.

THE St. Nicholas Nurse and Loan Society is the centre of much good work throughout a wide district. It dispenses aid to the sick poor by supplying nurses, providing food from the invalid kitchen, and sending convalescents to homes at Shotley Bridge and Hexham for change of air. The number of cases attended last year were 2,157.—Miss Marion Hunter, niece of Sir William Hunter, has accepted the appointment of lady member on the Commission to Inquire into the Plague in India.

### Lectures to Surgical Nurses.

By H. A. Latimer, M.D. (Dunelm), M.R.C.S. of Eng., L.S.A. of London, Consulting Surgeon, Swansea Hospital; President of the Swansea Medical Society; Lecturer and Examiner of the St. John Ambulance Association, &c.

### XVI.—STHENIC AND ASTHENIC FEVERS—THE "TYPHOID STATE"—NURSING IN PRIVATE HOUSES.

In my last lecture I drew your attention to the fact that fevers were spoken of as being sthenic, or strong, and asthenic, or weak, which means, in plain English, that they are either high or low in point of symptoms. When dealing with a strong form of fever we expect to meet with a full bounding pulse, delirium, or much brain excitement, and the exhibition of much strength by the patient in his movements; and in a low form of fever the reverse condition of affairs prevails. In low forms we are often brought face to face with a train of symptoms of the gravest kind, and the depressed condition of the system which they indicate is spoken of as the "typhoid state." You must not confound this word typhoid as used here with the specific fever which is variously known as typhoid or enteric fever, for it refers to quite another state of affairs, and one which may arise during the prevalence of any kind of illness which is marked by a profound depression of the system. The word typhoid means "like to typhus," and because in typhus fever, which is a profoundly depressing and prostrating disease, symptoms like these which I am about to detail to you are wont to set in, the word was coined to aptly describe a like condition when met with at any time and in any variety of illnesses. It is a very useful term for our purpose, and when used in this sense carries a large amount of meaning with it, although it is so short. The patient has fallen into "the typhoid state" will mean something like this. A person who has been suffering from one of the surgical fevers I have been telling you of, or from an inflammation of the lung, or from any condition whatsoever of disease, becomes quite prostrate in bed. If he has had well-marked symptoms of the disease afflicting him, it is noticed that these symptoms are no longer prominent ones, or that they are changed in character. Hitherto he has been able to maintain his position in bed, but now he slips down into it, and lies "all of a heap," as the muscular strength he formerly possessed has left him, and he no longer has the power even to lie in a comfortable manner. He ceases to complain of his ailments, and lies muttering to himself: and he also, if left with sufficient strength, is apt to fidget about with the bedclothes, or even to be industriously putting out his hands to catch and pick up imaginary things in front of him. His pulse becomes very feeble, and very little pressure upon it with the finger suffices to stop the wave of blood there. The tongue becomes dry, like leather, and is very apt to crack in places, and a brown, dirty scum collects about the teeth and mouth. Twitchings of muscles take place, and when you are feeling the pulse you will notice that the tendons of muscles at the wrist start up from time to time and are "on the work," as it is called. As he has ceased to take notice of outside things, he allows urine to collect in his bladder, and, being very prostrate, his flesh breaks down easily wherever pressed upon, and bed-sores form with great rapidity. When the condition has continued awhile he may fall into an absolutely apathetic state, during which he lies on his back with his eyes wide open, but evidently seeing nothing—shut off, in fact, from the world and all that is going on around him. When you see such a train of symptoms as the above the condition of the patient is most critical. He need not necessarily die, but he is in the greatest danger of doing so. Some of your greatest triumphs will be in the recovery of people from such a state, for much depends, if the patient is to get well, upon your labours and attention to him. What these attentions shall be I will tell you presently in the course of the remarks which I am about to make to you on the subject of the general nursing of fever cases.

You will readily appreciate the fact that nursing patients in hospitals and in private houses are two very different things. In the first case the work is carried on in buildings which have been especially erected for the care of the sick, and, this object having been borne in view by their designers, everything has been adapted to the point of view of obtaining the best results in that work. All things concerned with the difficult questions of ventilation, warming, drainage, and cleanliness of the buildings have been carefully considered, and the general result has been that hospitals of the modern type are generally adapted for the end their promoters and supporters have in view. In the case of private houses the reverse often obtains. Drainage is frequently faulty, and with difficulty can the great necessities of proper ventilation and warming be carried out in many rooms which have to be chosen for the purpose of nursing sick people.

If an opportunity is afforded you of making a choice of rooms in a house, choose one which has the sun shining upon it in the afternoon and evening, and let it be sufficiently large to give you plenty of fresh air. See that the fireplace is well situated, and that the chimney will draw well when a fire has been lit. Remove carpets, unnecessary hangings to the windows and bed, and all stores of clothes packed away in cupboards or chests of drawers in the room, so that the germs of disease, which cling readily to material of this kind, may have no abiding place. Ensure a thorough cleaning and scrubbing of the walls and floor; hang up a thermometer in some place where it will not be affected by the heat of the fire or by draughts of air, and, throughout the illness, keep the temperature at about 60 degrees Fahrenheit, not allowing it to mount above 65 degrees. Much of your comfort in dealing with the invalid, and of his, too, will depend upon the bed in which he lies. You have a good model of what is the right kind to be chosen in the beds which are used in our hospital, and in all other similar establishments. A single bed about 6 ft. in length by 3 ft. is the right size, with a mattress on it. With a bed of this sort you can easily move a patient without straining yourself, and with such a firm hold of him that he will be more comfortably moved and lifted than would be the case were it a larger one. Moreover it takes up a minimum amount of room space, which is a great comfort for every one concerned. Do not be afraid of fresh air admitted from without through the window. You cannot well have too much of it as far as the patient is concerned, for all such people give off harmful products from the skin, lungs, and other excretory organs, which require free dilution with fresh air if they are to get well from illnesses. This fact is taken into consideration by the Poor Law Board, who require that the dormitories in workhouses shall allow 1,200 ft. of cubic space for lying-in cases and offensive sick; 850 ft. for the sick; 700 ft. for the infirm—same room night and day; 500 ft. for the infirm, with separate day-room; and 300 ft. for the healthy. I quote these figures to emphasise what I am saying about the abundance of fresh air which should be provided for all sorts of invalids.

### Post-Graduate Clinics for Nurses.

By a Trained Nurse.

### XXXIII.—HOT AND COLD FOODS.

It is no small matter in the dieting of the sick—especially during long illness—that the palate as well as the stomach be fed. For if the palate tire of a particular food, be sure that the mysterious sympathy existing between palate and stomach will affect the proper disposal of it. It may not be easy to account for the fact chemically, but no less is it a fact that food pleasing to the taste is more readily and easily assimilated. This careful selection by the palate of different kinds of food at different times is too apt to be dismissed under the generic term of faddiness about food. As an illustration of what might come under the heading of "fads," I may cite the cases of those patients who declare they have no difficulty in digesting milk, and that all sensation of heaviness is taken away by a small lump of sugar taken with each glass. I have constantly met with this idiosyncrasy in patients suffering from all kinds of illness complicated by gastric difficulty. Such patients aver that proper digestion of their food is quite out of the question unless they take a small quantity of sugar with or after each meal. And I have frequently found that a tiny sweetmeat, such as a caramel or a piece of sweet digestive candy, seems an actual necessity towards the digestion even of beef tea. I have questioned many doctors on the subject, and they say it is one of those peculiarities of digestion difficult to explain, but all agreed where a patient exhibited a natural craving for something sweet—unless, of course, there were special reasons in a particular case where this might be harmful—that it was as well to yield to the patient's importunity. So I mention it here since it may prove of value as an addition to the dietetic knowledge of nurses. And yet, though this seems such a trifle to the onlooker, and might be dismissed as the faddiest of "fads," it is a trifle which may make all the difference to the patient not only so far as the digestion and the nutrition afforded by his food is concerned, but also in his gastric comfort.

The nurse, too, should study the delicate science of discerning whether hot or cold foods are more likely to be acceptable in certain conditions and at certain hours. It is not difficult here to establish a general principle that in cases accompanied by lowered vitality and in illnesses whose effect is depressant, hot foods will usually be more acceptable than cold. In cases of influenza, for instance, with its depressant symptoms and in spite of accompanying fever, the patient usually craves hot drinks, for he feels that his lowered tone can hardly sustain a gastric cold douche. Just in the same way as persons with poor circulation and low nervous energy have an instinctive prejudice against a cold bath, so under similar conditions sick people usually take kindly to hot drinks as being more stimulant than cold.

Very few persons—even in robust health—have the inclination at their first morning meal, unless the climate be very hot, to take cold drinks. After the long fast there is an instinctive desire for a cup of tea or other hot fluid. This principle of reasoning carried into sick-room diet will be of infinite value. Only it is well to bear in mind the exceptions—they are very important—in fact, so important that I am tempted to alter a familiar proverb and convert it into sick-room use, "Take care of the exceptions, and the rules will take care of themselves."

The exceptions to the generalities of hot and cold feeding lie in those common cases of nausea and vomiting—which may occur in the course of all types of illness—usually relieved by ice cold food and small pieces of ice swallowed whole These are familiar instances. On the other hand, when ice-cold remedies fail to relieve nausea and sickness, small quantities of extremely hot fluids will sometimes act like a charm. These are the exceptions, and it is well to remember as a general principle that ice-cold food and drink is not acceptable to the aged or to patients with weak hearts and poor circulation. In various heart diseases hot drinks are most acceptable, only it sometimes happens that the distressing nausea and vomiting attendant on some forms of heart trouble are readily relieved by ice, so that while cold substances may act as further depressants, they become essential by virtue of their local effect on the digestion.

In cases of nausea and vomiting, whether temporary or chronic, besides watching very strictly over the diet, the first nursing step to take is to at once put on a wide flannel binder, coming well up under the arms and well down over the abdomen. Vomiting very frequently results from a gastric chill, but whatever the cause may be the flannel is most helpful. I find so often in private practice that these simple flannel binders—perhaps one of the most useful nursing adjuncts we possess—are rarely, if ever, applied. Yet from the typhoid fever patient down through every varying stage and sort of diarrhœa, to the slightest gastric complication, there is perhaps nothing which gives such immediate and ready help as a yard or two of flannel. Unfortunately these useful little remedies and simple aids are not taught in hospitals, and I often call to mind the patients of my old training school, suffering from constant vomiting and with all sorts of abdominal chills and diarrhœal complications, who were clad only in a cotton nightgown—as often as not open all the way down the front—and with no flannel binder to comfort the congested condition of stomach and intestines. Sometimes a flannel jacket was furnished. In most of the children's hospitals such nursing points as these are attended to with scrupulous care. But one certainly does not learn the niceties of nursing in a routine ward of adults. In cases of nausea and vomiting, in addition to the dieting, the nurse may in private practice and on her own responsibility try a hot turpentine stupe or poultice. And it is a valuable little wrinkle to know that in many cases of sickness a little burnt brandy will be retained far better than the raw spirit. A dessert spoonful of good brandy, set alight by a match, added to a wine-glassful of cold whey or barley water, though the whey is preferable, and administered in tea-spoonfuls at intervals, is an excellent means of administering some nourishment in a form which most probably will not be rejected.

Another nursing point which should be specially attended to in cases of nausea and vomiting is that, since cold and lowered vitality are apt to prove contributory causes, it is important to keep up the patient's heat by artificial means. It would not occur to every nurse—and yet it is a fact easily learned in practice—that a hot-water bottle to the feet has a distinct effect on gastric trouble, and this simple expedient frequently relieves persistent sickness and nausea. It is just these complexities and complications which have made nursing into an art. Were nursing a matter of routine, a lay figure worked by wires could be constructed to administer food and medicine at stated intervals. But it takes a trained nurse to know when she may with advantage go out of the "beaten track," and apply in the right way the varied knowledge she has gained while studying her calling.

### Nurses and Club Life.

By HELEN FOGGO THOMSON.

CLUBS for women may still be regarded as a new or a modern departure, but they are already recognised as being a necessity, and it is evident they have come to stay. Professional women, more than any others, have discovered their utility, and of all professional women trained nurses most require some such provision. But though nurses are to a great extent agreed as to the desirability of starting a club for themselves, it is quite an open question whether they yet understand exactly how to use one.

A vague notion seems to have got abroad among some people that the club is a sort of institution whose members are bound by rigorous rules and restrictions, and where liberty of action is almost unknown.

Anyone who knows the Victoria Commemoration Club for Nurses, in its handsome and convenient premises at 29, Southampton Street, Strand, will know that this impression is erroneous. There are rules, of course, such as there must be and are in all clubs, but they are made simply in the

A CORNER IN THE NURSES' CLUB.

interests of the members, and are in no sense of the word restrictions. The place is really and truly a club, and nothing else; that is to say, it is a place in which the members may meet for rest, for talk, to read the papers, to write letters, to entertain their friends, and where they may have either breakfast, luncheon, tea, dinner, or supper, properly served at a moderate price. The subscription entitling a member to all these benefits comes to about three-farthings a day—less than the price of a postage stamp or a morning paper.

The club was opened in February last, and there are now close upon 300 members, but it is felt that this is not a reasonable proportion of the 15,000 or more nurses in the United Kingdom. Nothing can be more desirable for the improvement of the *esprit de corps* of the profession than a neutral centre of this sort.

There seems to have been a mistaken impression prevalent at one time that a club for nurses might in some way undermine the authority of the matrons of hospitals and nursing institutions. This is an absolute delusion, founded entirely on misconception. The only results that can follow from the success of such a club are the greater enthusiasm of the nurses for their profession, and an increase in their own health, happiness, and comfort. Indeed, when a member has paid her subscription she has really another home, with many advantages which only a very elaborate private home could offer her.

In one respect, and one only, does the club fall short of a home. At present there is no sleeping accommodation. An effort is being made to supply that want, but before this can be done an increase in the membership is necessary. Already breakfasts and suppers, which were not supplied when the club first opened, have been demanded, and as many members come up from the country the necessity for bedroom accommodation is obvious.

It is clear, therefore, that nurses by joining now will do the club a good service, for this, as the pioneer club, is deserving of all support. It is equally clear that by joining they will be benefiting themselves, for those who join now will be admitted at the present subscription of one guinea, and will always continue to pay that amount, even though it should in time be thought necessary to raise the subscription to new members. It is further proposed to allow nurses to join for the remainder of this year for a payment of 5s., without an entrance fee, in order to enable nurses to test the advantages and comforts of the club for three months, and if, at the end of that time, they think fit they may continue their membership.

In spite of its low subscription, and in spite of the very reasonable prices charged, the Victoria Commemoration Club can compare favourably so far as comfort and general advantages are concerned with any ladies' club in London. The meals are such as members can place before visitors of any rank in life with confidence. The cook is a treasure, and those who know club life know how much depends upon a cook. The whole place is bright, cheerful, well-furnished, and ideally situated. All that it now needs is more members and bed-rooms, but without more members bed-rooms cannot be added. It is felt that when the great body of nurses in the United Kingdom have these facts brought before them many of them will join for their own sakes, and many more still for the sake of their profession.

### Appointments.

### MATRONS.

COTTAGE HOSPITAL, AXMINSTER, DEVON.—The post of Matron of this hospital was given to Miss A. M. Stewart Crichton on September 25th. This lady was trained at University College Hospital, London, and she has since had considerable experience in various branches of nursing, her last appointment being to the Hospital for Sick Children, Pendlebury.

ORIOLET COTTAGE HOSPITAL.—Miss Clara Bowles, who received her training at Charing Cross Hospital, is the new Matron of this hospital. She has held the position of matron to three cottage hospitals successively, viz., Sidmouth, Winchcombe, Halstead, and the Victoria Home for Nurses at Bournemouth.

CITY OF COVENTRY FEVER HOSPITAL.—Miss Margarette Davidson was appointed on October 12th Matron of the above hospital. She was trained at Bradford General Hospital and Leeds Fever Hospital, and has been charge nurse City Hospital, Liverpool, and charge nurse Fountain Hospital, Tooting.

CREWE ISOLATION HOSPITAL.—Miss Elizabeth B. Norris has been appointed Nurse-Matron of this institution. She was trained at Worcester General Infirmary, and has subsequently been charge nurse of Cambridge Infectious District Hospital and sister at Birmingham Fever Hospital.

### On Preparation for Operation in Private Houses.

By E. STANMORE BISHOP, F.R.C.S.Eng., Hon. Surgeon, Ancoats Hospital, Manchester.

(*Continued from page* 15.)

WASH BASINS.—Four large wash-hand basins will be wanted. One for washing the surgeon's hands, one for clean sponges, one for dirty sponges, which should have blood, &c., rinsed out before replacing amongst the clean ones, one to contain solution for occasional use during the operation.

DISHES FOR INSTRUMENTS.—One or two flat dishes for instruments.

EMPTY PAIL.—A large pail or foot-bath for dirty water, contents of ovarium cyst, &c.

BASIN FOR SUTURES, &C.—Some surgeons prefer to have their sutures, ligatures, &c., in a small basin filled with some antiseptic solution.

TOWELS.—You will need plenty of towels, at least four of which should be smooth. These four are soaked in antiseptic solution, wrung out, and are to be placed around the operative area, so as to prevent instruments, ligatures, &c., from touching the blankets. But you will probably want at least a dozen towels altogether. Of course I need not insist upon their being absolutely clean.

MANY-TAILED BANDAGES.—When the operation is over and dressings applied you will want two many-tailed bandages, and now is the time to make them. The best way, I think, is to get a single piece of soft flannel wide enoughto reach from the patient's epigastrium to a point two inches below the pubes and long enough to go one and a half times round her. Tear the sides at points two inches and a half apart down towards the centre, leaving a space the width of her back between the two rows of tails. Cover this with lint, the soft fluffy side uppermost, and tack it on. This is better than tacking strips together over a central strip, because in this way no ridges are left to irritate the skin. Of course, only one is required at a time.

OPERATING TABLE FOR TRENDELENBURG POSITION.—Should the Trendelenburg position be required, it can be obtained in the following way: Get a stout chair, of the kitchen chair kind, and if possible with a strong cross-piece between the two back legs at their extremities. If this latter addition is not to be had, turn the chair upside down and fix firmly a strong double piece of clothes line across. You will see the use of this directly. Attach four pieces of rope, each eight feet long, by their centres to the following points: One to each corner of the upper rail of the back of the chair, and one to each front corner of the seat, knotting them firmly and leaving both ends of each rope of equal length. Now invert the chair upon the table, so that the top rail of the back will lie close up to and parallel with the pillows at its head. The chair should rest upon the table by its top rail and the front edge of its seat. Tie the two ropes attached to its top rail to the legs at the head of the table, and those attached to the seat to the two lower legs. Pull all tight, and knot the ropes in this position. The back of the chair will slope upwards from the pillows, and the legs of the chair will point obliquely towards the ceiling. Cover the back of the chair with a bolster placed lengthways. When the patient is placed upon this, her head will rest on the pillows, her back from the shoulders down to the hips and the back of the thighs to the knees will rest upon the back of the chair and its back legs, and her knees will be supported by the cross-bar or rope which you have placed between the lower extremities of the two back chair legs. Her legs will hang down between the legs of the chair, and should be secured by a clovehitch taken in a bandage, the ends of which are fastened to the front chair legs near the seat. You will see the necessity for making that top cross-bar of wood or rope strong and firm, since it sustains much of the patient's weight. (See Fig.)

HOT WATER BOTTLES.—When the patient is in bed again she will probably require three or four hot water bottles. See that these are ready. If the usual kind are not procurable good substitutes can easily be made out of old whisky or wine bottles. See that their corks fit accurately, and they are all the better if covered with flannel.

CAGE.—To keep the weight of the bedclothes from pressing upon her a wire cage is wanted, but if this cannot be had, a good substitute can be made out of an old circular bandbox by knocking out the bottom, taking away the lid, and cutting down the centre of one side. When opened out somewhat and placed transversely over the body it forms a very good bridge, upon which the clothes may be supported. If the ends tend to slip outwards they can be prevented by placing them between sandbags made out of old long stockings, which are filled with either salt or sand, and their ends sewn up.

By going over your prospective work in this way you will see that you have produced a list of the things you will want in the order in which you will want them. And now is the time to see that they are all available.

THE FIRE AND THE TEMPERATURE OF THE ROOM.—On the morning of the operation you will thus have everything in readiness except to see to the fire and the temperature of the room, which for an abdominal section should be about 80 deg. to 85 deg. F.

HOT WATER.—The hot water should also now be prepared by boiling for 15 minutes and kept as already directed. Altogether from five to six gallons of water should be in readiness.

COVER THE INSTRUMENTS.—See that after the surgeon's preparations are made, and before the patient enters the room, that everything is carefully covered.

Your duties during the operation are, of course, the same as those in a hospital, but under these new conditions you may have to do yourself all that is here done by two or three. Make it your business whilst here to thoroughly understand what each nurse is doing, so that you can without difficulty take her place. Above all things remember that the success of an operation depends upon the asepticity —that is, the complete absence of germs from every one who touches instruments, sponges, or dressings, and so first sterilise your hands as carefully as does the surgeon, and then if during the work you should happen to touch anything in the least doubtful—dress of patient, blanket, cord of irrigator, door, table, anything—soak your hands again thoroughly in some reliable antiseptic before handing sponge, ligature, or instrument. A nurse is as powerful for harm as a surgeon, and the whole thing may be a failure simply because you are not sufficiently careful. I confess I greatly prefer to see a nurse remove those dainty white cuffs, of which she is so proud, and turn up her sleeves—usually much too tightly fitting—whilst she scrubs arms and hands as thoroughly as the surgeon himself. What should we think nowadays of a surgeon who operated in his frock coat, and let his coat sleeve touch patient or dressings? Some people seem to imagine that the surgeon removes his coat and turns up his shirt-sleeves for fear of dirtying them, and not because he knows that they might be a potent cause of danger from carrying spores and bacteria to the wound.

As soon as the operation is over and the patient in bed, carefully packed round with hot bottles, if required, set to work at once to remove all traces of the morning's work, so that when the patient recovers there may be no operating table—not even a soiled sponge or a basin of bloody water remaining. These things seem nothing to us, but to an unaccustomed mind they are veiy revolting, and may start vomiting you will find it take all your time to stop.

When the patient recovers consciousness, cover her face lightly with a shawl, the counterpane, or anything else light and warm, and open the window top and bottom, as far as possible, for three minutes, so as to empty the room entirely of chloroform vapour, and then close it. Repeat this about twice a day, and your room will never have that horribly stuffy, unhealthy smell which characterises so many sick-rooms, and militates so strongly against the chances of recovery of its unhappy inmate. Do not, I beg of you, follow that time-honoured but abominable practice of "only opening the window the least little bit," causing a cold breath of air to play continuously on some unprotected part of the patient's body, which, by its contrast in temperature, to that of all the covered position, is the readiest way to induce a chill of any I know. It is so potent a method of producing catarrh that I wonder no one has patented it.

If your surgeon should forget, remind him to forbid the ingress into the room of anyone but yourself and one or two more to be distinctly specified; and see that this order is carried out. Permit no one to stop gossipping, crying or laughing immoderately. One more hint as to yourself. Whilst feeling and showing the utmost sympathy for your patient, do not permit yourself to be too familiar. I know an extremely good nurse who utterly ruins her chances by always calling her patients "Dear," "Love," "Darling." Such names are all very well under certain circumstances—you may know better than I when they are suitable—but they are distinctly out of place between nurse and patient, and may be most strongly resented by the latter.

The after treatment differs little from that to which you are accustomed here, except that you will probably have to share some of your duties with some unprofessional member of the family. Do not entrust to them any of the really technical duties of a nurse. Always pass the catheter yourself; take the temperature and pulse yourself; do all needful dressings yourself; all necessary washing and sponging yourself; look after the temperature of the room yourself; the ventilation yourself; all enema-giving yourself; and simply leave to others the giving of food and the needful watching required while you are away.

Lastly, remember that the house in which you are staying does not belong to you. The mere fact that you are the nurse duly appointed by and responsible to the surgeon in charge does not give you a right to assume the entire control of the building and everyone in it any more than the fact of being the surgeon in charge would justify the operator in assuming a right to govern the household. In certain things, and for the definite purpose of getting your patient well, you are quite right in expressing your opinion; it may be a very decided one on matters germane to that object. You will ordinarily find that everyone is only too glad to be guided as to those things by one who they clearly see knows more about it than they, especially if they realise, as soon they will, that pure anxiety for your patient's welfare dictates your action; but should they be stupid or unreasonable, it is better to yield and simply report to the surgeon than to create any disturbance by high-handed proceedings at the time. What can be worse for your patient than a vulgar row, or sullen carelessness on the part of those by whom you are surrounded and upon whom you have mainly to depend for her, and for your own, comfort. But it is not to such things as these that I refer. I have known nurses go into a private house, insist on choosing their own bed-room, even taking that of the mistress of the house, ordering special meals for themselves at peculiarly inconvenient hours, and generally treating the whole house and everyone in it as though they existed simply for the nurse's benefit. Such nurses forget that thereby they simply prove their relationship to the immortal Betsy Prig and Sairey Gamp, whose callous indifference to anything but their own comfort was the main article in the indictment drawn by Dickens against them, and which held them up to the contempt and ridicule of all the civilised world. I feel sure that it is only necessary to draw your attention to this, nor do I imagine that any of you are likely to act in this way; but I have seen this sort of thing occur, and it cannot be too clearly understood by the general public that there is nothing in the training of hospital nurses which countenances such behaviour, and that the great bulk of such nurses, as well as those who train them, utterly repudiate it.

### Our American Letter.

GREAT pains have been expended over the preparation of the plans for the new Albany Hospital buildings, New York. Two of the medical staff visited similar institutions in different parts of the country, and both caused plans to be drawn which were submitted to the Building Committee. Those prepared by Messrs. Fuller and Wheeler from the instruction of Dr. Vander Veer have been recommended for adoption. They show eight blocks of buildings two stories high. One contains offices for administration, one kitchen, one operating theatre, and one nurses' quarters; the remaining four the wards, arranged pavilion-fashion, running parallel at right angles to the main corridor.

The opinion of Dr. Johnson, Governor of the State Board of Charities, is awaited with some anxiety by the well-wishers of the proposed new hospital at Butler (Pa). By the energy of the Ladies' Auxiliary Committee about £2,000 has been raised for building purposes, besides a gift from Mr. Caragie of £500, and it is hoped that the Legislature will supplement these sums by £1,000 if Dr. Johnson does not veto it.

The Graduated Nurses' Protective Association of the State of New York is the name of a new society which may become a nurses' trades union. The first aim of the promoters is to protect the trained nurse (any certificated nurse of New York may become a member), and then "to elevate the standard of nursing generally, and to promote unity and goodfellowship amongst the members." It will found and maintain a hospital for sick graduate nurses, a home for aged and infirm, and in connection with both a post graduate school, where nurses on completion of their general training may take up special subjects. The association is founded by a trained nurse and masseuse holding a Swedish certificate, and announces its intention of applying for legislation regulating the practice of massage. This determination has provoked considerable opposition to the whole movement. The weak points in the scheme are the narrow sphere to which it proposes to confine its operations, viz., New York City, and secondly that none of the heads of the more prominent training schools have countenanced it.

In Baltimore the Johns Hopkins Hospital has had additional accommodation for the nursing staff provided by fitting up rooms lately occupied by domestics in the Apothecaries' building for their use. The rooms answer the purpose excellently, as they are large and well constructed. Thirty-four nurses are thus housed, but the ight nurses' quarters have still to be arranged for.

### The Nurse and Her Savings: A Dialogue.

*Scene:* A Bank Parlour. *Present:* A Hospital Matron and a Banker.

A HOSPITAL MATRON: The fact is I am greatly worried by my nurses and their money matters. They are always asking me what to do with their money. To you it may seem a great deal of fuss about very little, but to them it is everything; it means the savings of their life—all that they have to trust to to keep them from the workhouse in their old age—and I assure you it is a great anxiety to me. They are so easily led astray by promises of high interest, instead of thinking of safety in the end.

BANKER: But have you not got a National Pension Fund?

M.: Oh, yes; but you see nurses always have such a lot of friends who will pester them with advice and promises of great returns and high interest.

B.: Dear me. No one ever offers me high interest; at least, no one I should like to trust.

M.: Ah! that's just it. It's just a matter of confidence. They trust all sorts of people. No sooner does a nurse begin to put a little money by in the Pension Fund than she tells some one or other about it, and as soon as it gets abroad that she has a little money then the harpies are down upon her with promises of all sorts.

B.: But what sort of people do you mean?

M.: Well, you see, some nurses are very good women, religious women, you know, and they get led into lending their money to chapel funds and school building funds, and all sorts of things, for which they are promised 5 per cent. interest, and no doubt they get it for a year or two. But a great deal is lost in the end. The fund gets used, there is no legal claim on any individual, it is not worth while going to law, and it is all lost. Then another set, often the cleverest, get taken in by the promises of high returns from starting a nurses' institute. Some specious woman, who says she is a good housekeeper, persuades three or four nurses to join her in starting a "nurses' institute." After saving for a time, and getting a good start towards a pension for their old age, they get tempted to draw their money out of the Pension Fund, and sink it all in one of these wretched "homes" or "institutes." The result is that they are practically tied fast, and go on working year after year, all their earnings go to keep up the home and pay the lady superintendent, and in the end the bailiffs come in and take everything. It seems next to impossible to make nurses see that for success an institute must have a large number of nurses to share the working expenses, and there are always persuasive women about to tempt them to throw their savings into ventures of that sort.

B.: Yes, certainly that is a pity.

M.: Now there is another thing, and really it is that I want to talk to you about. You know our Pension Fund?

B.: Yes.

M.: Well, you see, nurses are being told that it gives a very poor return, and are being asked to insure in other companies.

B.: But surely nurses ought to be able to see that a Fund like that, managed by some of the best and most trusted men in the city of London, must yield them as good a return as it is possible to earn, with safety that is.

M.: Yes, they ought to see it.

B.: Then it is plain that whatever money the Fund earns the whole comes back to the nurses. There is no interest or dividend to pay to outside shareholders, there are no directors' fees, no commission to pay to collectors and agents. The money is invested to the best advantage, it is all under trust, and the whole of the funds and profits come back to the nurses in interest or bonus. No commercial company could possibly offer such terms as these, nor nearly as much in fact. It is notorious how much goes in expenses of management and collection in competing insurance companies, especially in those that deal in small sums. Why, there are some that spend upwards of 30 per cent., that is, fourpence goes in expenses out of every shilling collected; or in other words, a payment which brings in a pension of £20 a year might bring in £30 a year if the company were managed with as little expense as the National Pension Fund is.

M.: How is it then that any company is able to offer the same pension beginning at the same age for a smaller annual payment than the National Pension Fund asks?

B.: Well, that is a very curious story. It is very like going to a shop where they offer you tea for less than cost price. You know there is something mysterious somewhere. Of course it does not matter with tea, because you drink it as you go on, but with an insurance for a pension it is another matter, for if anything goes wrong with the life savings of a nurse it often means the workhouse.

M.: Then you mistrust a company which asks a lower premium than the National Pension Fund does?

B.: Well, it is certain that such a company cannot offer you the same advantages as the Pension Fund, and at the same time charge a lower or even as low a premium, as it pays dividends to shareholders, directors' fees, and other expenses, from which the Pension Fund is exempt, and all of which must come out of the premiums.

M.: But the question put to me by one of my nurses is this: If a company chooses to take nurses at a lower rate, even at the risk of making a loss by doing so, what is that to us? Why should we not take advantage of the offer?

B.: You should consider how the risk of loss is counterbalanced. A company, which for the sake of getting into its hands the business of insuring nurses offers them rates which will not pay if all pensions insured mature and become payable, may be relying on forfeited premiums to make up the loss. Nurses who insure in the Pension Fund run no risk of forfeiture of premiums if, when unable or unwilling to continue the payment of premiums, they avail themselves of the opportunities of withdrawing what they have paid in. The Pension Fund is quite safe, and returns as good an interest as a safely conducted business can do. But I wish you nurseswould not trouble so much about the question of the nominal return on your money.

M.: Why not?

B.: Because the nominal return does not a bit matter if they are insured with the Pension Fund. Whatever interest and profits are earned by their money, except something over 3 per cent. for office expenses, comes back to them in the shape of bonuses, or interest, or pension. They get it all, whereas in a company the shareholders get it. Why, a company which is now offering low rates to nurses is earning over 50 per cent. interest for its shareholders, and its expenses amount to over 45 per cent. of what it receives, while the National Pension Fund only spends between 3 and 4 per cent. of its income from premiums for the same purposes, and the remainder the nurses will receive as a bonus.

M.: Do you mean to say that, out of every £100 received in premiums of every kind by the company you are speaking of, more than £45 go in expenses?

B.: Yes, that is about it. But there is another side to the question which nurses should never forget, and that is that the advantages offered by the National Pension Fund are so much greater than any company can give. Not only is there the fact that the nurse's money is not used to pay directors and to provide dividend for shareholders, but she gets advantages that are not offered by any company. The first of these is that she can always get her money out with compound interest added.

M.: Yes, that is a great advantage. How often nurses get married or drop into some other sort of occupation. It is very nice being able to get all their money out with interest.

B.: Of course it is. Practically, a nurse may look upon the Fund as one of the best and simplest ways of investing her savings, quite independently of the pension. She can either draw out what she has put in or she can will it in case of death, in either case obtaining compound interest at the rate of 2½ per cent. all the time. Now the insurance company I am thinking of, which I know has attracted the attention of nurses lately, will only return her, on withdrawal, 15s. out of every pound she has paid in, and in some cases only 10s., and that without interest. These forfeits, which are a dead loss to the nurses, are a source of income to the company. The forfeiture is a matter of business.

M.: But I am sure the Pension Fund does not do like that.

B.: No; certainly not. The very object of the Pension Fund is to help nurses, not to make a profit for shareholders.

M.: That is what I have always found. I am sure, from what I have seen, that the managers of the Fund extend every consideration to nurses, and do not cancel the contracts.

B.: I do not think that nurses understand as they ought how largely the pension which they will receive must be increased by the bonus which will be added every five years without any increase in the premium to be paid by them. Not only so, these bonus additions will go on when the nurse ceases to pay, so that even after she enters on her pension the amount she receives will still go on increasing every five years until her death. You see, when nurses talk of a cheap premium they ought to inquire what they get for it.

M.: Then, of course, one must add the fact that the National Pension Fund is a sick fund also, which is not the case with the competing companies, besides which, in case of a member of the Pension Fund falling into distress, there are the benevolent funds, which are under the independent administration of hospital matrons, and of well-known ladies who take an interest in nurses, so that, besides her pension, a member feels she has friends who will and can help her in case of need.

B.: Yes. The fact is there is no comparison. With the company a nurse might pay a slightly smaller premium, of which, however, a large proportion would go in management; a further proportion would be invested to secure the pension, and the remainder would be divided amongst the shareholders as profits. With the Pension Fund, on the other hand, between 3 and 4 per cent. covers the whole working expense, and every penny beyond that is returned to the nurses; not a penny goes either to shareholders or directors. Again, a considerable amount of money has been given out and out to the Fund, all of which adds both to the security offered and to the amount available for distribution as bonus. The Fund also has the advantage of being managed by the leading financiers in London, including the Rothschilds and the most trusted City men; it is incorporated under the same Acts of Parliament as the companies are, and is conducted under the supervision of the Board of Trade, just the same as an insurance company; with this great difference, however, all the profits go to the nurses instead of to shareholders and directors. Besides, although it is founded specially for providing pensions, it can conduct life insurance business too, and it is so constituted that all the money paid in by a policyholder, with compound interest added, can be drawn out, or can be willed at death. I do not know what more your nurses can want. For, after all, the great thing is security for the future and an adequate provision against sickness and old age for the whole of life. These advantages can be secured with assured certainty by joining the Royal National Pension Fund, and no company can compete with it, as a matter of business, by offering to nurses or anybody else, equally good, much less better, terms for the same benefits.

### The Royal British Nurses' Association.

THE meeting convened by the so-called "Members' Rights Defence Committee" of the Royal British Nurses' Association, which we announced in our last issue, was held at St. Martin's Town Hall on Wednesday, the 13th inst. On a liberal estimate about 120 persons were present, amongst whom were Mrs. Bedford Fenwick, Miss Margaret Breay, Miss Beatty, Miss Homersham, Miss Balgarnie, Mrs. Somerville, Mr. George Brown, and Dr. Bedford Fenwick. Besides the Press there were only about half-a-dozen men present, and nurses in uniform formed a very small percentage of the remainder, who, we understand, were principally members of the public, and not of the association. Dr. Hugh Woods took the chair, and was supported on the platform by Dr. and Mrs. Bedford Fenwick.

The CHAIRMAN, having read at full length the two "protests" which had been so extensively circulated, called upon

Mrs. BEDFORD FENWICK, who moved the first resolution, which was as follows: "That this meeting expresses the astonishment which is widely felt that the officials of the Royal British Nurses' Association have not demanded a public inquiry into the grave charges made against their management, because such a reluctance on their part admits of only one explanation. In view of the great public and professional interests which are involved, this meeting considers that such an inquiry is imperatively needed, and it therefore hopes that all members of Parliament will support a motion for the appointment of a select committee, which will be made next session in the House of Commons." The speaker prefaced her remarks with an apology for reading what she had got to say, but she did so in self-defence because much that she said in public, and a great deal which she never had said, was systematically misrepresented, especially by one organ in the press. In too great a hurry to stop to explain how what she had never said could have been misrepresented, Mrs. Bedford Fenwick proceeded to read her statement, in which she accused five medical men, to wit, Sir James Crichton Browne, Mr. T. Pickering Pick, Mr. John Langton, Mr. Edward Fardon, and Dr. Bezley Thorne, generally of mismanaging the association. Speaking of their financial management, she said that through this mismanagement the association had become "the financial dependents of the treasurer and his supporters, who naturally argue that those who pay the piper have the right to call the tune." Or as she also put it they were carrying into practice the old Russian saying, "Starve your mujik, and he will always remain a serf."

Miss BALGARNIE, who said she came there at Mrs. Bedford Fenwick's invitation, not as one who had any knowledge of the very onerous profession of nursing, but as a mere outsider, the very class of people that Mrs. Bedford Fenwick and the nurses now had need of, seconded the resolution.

Mrs. SOMERVILLE, another "outsider," supported the resolution, detailing her experiences at the last annual meeting of the Association, into which, being in the neighbourhood of the Imperial Institute, she had wandered in order to admire the nurses' costumes, and in the proceedings of which she, although not a member, had taken part.

Miss HELEN FOGGO THOMSON, a late secretary of the Association, pointed out that there were but few nurses present, and said that Mrs. Bedford Fenwick did not represent the Association—(applause)—and did not even represent the nurses. (Applause and hisses.) This meeting was called by Mrs. Bedford Fenwick in a fit of pique because she had been taken off the Council. (Cries of "Come on the platform," and interruption.)

The resolution was put and declared carried unanimously, although less than half those in the room voted for it, and some hands were held up against it.

A resolution to the effect that copies of the above resolution be sent to the members of the House of Commons followed naturally on the first resolution, and was agreed to.

Miss ELINOR PELL SMITH, referring to Miss Thomson's statement, said that she knew many nurses who trusted and believed in Mrs Bedford Fenwick.

The usual votes of thanks concluded the meetin .

### Novelties for Nurses.

### AUTUMN NOVELTIES.

We have received from Egerton Burnett (Limited), Wellington, Somerset, a large consignment of patterns which merit close attention. This renowned warehouse has long held its own for the high quality of its manufactures as well as for the excellence of its designs and variety of its colourings. Many new fabrics are included in the assortment before us, which surpass in beauty any we have hitherto seen. Especially attractive is a material in black, dark brown, or green, shot with silk of a contrasting hue, giving an impression of the coloured foundation so fashionable this summer. There are some charming designs in Winsey very suitable to the season, and we can strongly recommend a stylish check cloth called "The Edinburgh" for walking or travelling dress. The variety in tweeds and serges is simply unlimited, and nothing can be more neat and ladylike than the dark navy-blue serges in light or heavy make which form so important a part of the whole. The special advantage of these serges is that they are unshrinkable and will stand any amount of rough weather. It is worth while, therefore, employing a good tailor to make them up, or Mr. Egerton Burnett is prepared to send out costumes ready made on receipt of measurements. We notice among the patterns a special line of Scotch tartans, 44 inches wide, in a soft fine woollen material. These would make admirable linings for the golf capes still so fashionable among both sexes. Cricketing and boating flannels are exceptionally good value for the money; those in white are beautifully soft and will be found equally suitable for ladies' shirts and coats. For river wear these flannels are simply ideal. They are warm and at the same time sufficiently smart and cool looking. With a pink or blue surah blouse, worn either with or without a jacket, the effect is excellent, and with care should last through the autumn without cleaning. Nurses, however, will be interested more especially in the dainty selection of cottons and cambrics displayed. The grey zephyrs are very soft and pretty, and there are several small checks in different colours in the same material. Noticeable among these is a beautiful clear cambric suitable for caps. This is sure to become popular, as it will wash well in addition to preserving its softness of finish. Fine linen for aprons is another item deserving attention, while the selection in cloakings is one that alone will repay sending for patterns.

### NOVELTIES AT MESSRS. E. AND R. GARROULD'S.

A visit to Messrs. Garrould's popular establishment (150, Edgware Road) is always interesting, and never more so than at the present time. In the nurses' department, to which unremitting attention is given, nothing seems to have been overlooked, and several new and ingenious designs, both in uniforms and medical appliances, attract the eye of the beholder. In cloaks there is a large variety and every sort of shape to suit all tastes. We particularly admired the "Helena" and the "St. Alban," both of which are new and very smart looking. The sleeves fall loose from the shoulder, forming a sort of cape, and the cloak itself fits tight to the figure, which is clearly defined. The well-known "Angelus," however, still holds its own, and few cloaks come up to it as an ideal garment for a nurse. There is also a rather fascinating double-breasted coat with full sleeves fitting into a band at the wrists; a black velvet collar makes a becoming finish at the neck, and the cloth, which is of excellent quality, is waterproof. The price of this comfortable garment is two guineas, and is of never-ending wear. All these cloaks are made in light and heavy cloth, according to the season. A sweet little Marie Stuart bonnet, made of fine straw and trimmed with velvet, is sure to find many admirers. With a long silk gossamer fall, its price is 15s. 9d.; but the fall is an adjunct which we should advise nurses dispensing with, as it is not only useless and costly, but blows about in a very untidy fashion should there be any wind. Models 57 and 58 are both charming and fit comfortably to the head, besides being extremely becoming. The caps are very pretty, especially the "Abbeville" and the "Dovercourt," which latter is quite a new design. It has a puffed crown with a full quilling of valenciennes lace round the front, and ties in a bow under the chin. Passing into the boot and shoe department we were particularly struck by the comfortable yet neat shape these necessary adjuncts to the toilette possessed. Nurses who are constantly on their feet suffer more than most people from the continued strain, and Messrs. Garrould are to be congratulated on an excellent shape they have brought out called the "Ease at last." Judging from its appearance this shoe amply deserves its name, and we have no hesitation in recommending it in the strongest terms. The cost is 7s. 6d., which cannot be called expensive for a first-class walking shoe, which has the additional merit of being hand-sewn, thereby adding greatly to its softness and durability. The "St. Mary" is an excellent shoe for ward wear, and is an improvement on other models. The india-rubber heel-cup is firmly attached by a new process and is guaranteed not to wear off, which is a very great advantage, and will doubtless be fully appreciated. In dress materials for nurses Messrs. Garrould have a large assortment, and very beautiful many of the designs are; they must be seen, however, to be appreciated, and patterns will be sent post free on application. Luggage, especially for nurses who go long distances, is always a subject of anxious consideration, and a well-sized trunk capable of containing a fair amount of wardrobe and yet be neither bulky nor heavy, is a problem well worth solving. The "Overland" trunk in five different sizes, in prices varying from 21s. 9d. to 30s., which is seen in another part of these spacious premises, is one of the most convenient, lightest, and neatest articles conceivable, and one which no nurse should be without. It has the advantage of taking up but little room in a carriage or dogcart; indeed, it would easily slip under the seat of the latter, which is a great convenience in the country, where the station is usually a mile or two distant from the house. Messrs. Garrould have recently opened a tea-room for the convenience of their customers, which will no doubt receive the encouragement it deserves. There nurses can meet and enjoy a social half-hour over the cups and saucers; and close by, through the thoughtfulness of the management, are placed for their use certain books of reference, such as the Medical, Nursing, and London Directories, and "Burdett's Hospital Annual."

### The Victoria Commemoration Club.

There will be an At Home at the club, open to members and their friends, on Thursday, October 21st, from 4 to 6.30 p.m. Lectures: It is also proposed to hold the following lectures during the winter session: Burns and Scalds; Medical Nursing; Surgical Nursing; Mental Nursing; Obstetric Nursing; Fever Nursing; Throat Diseases; Eye and Ear Cases. Invalid Cookery: The cookery lessons will consist of a course of six, but on the other subjects there will be a single lecture only. Members and others desiring to attend are requested to send a post card to the secretary stating what lecture they propose to attend, and mentioning the hour and day of the week most convenient to them. Unless a sufficient number subscribe beforehand, the lectures will not take place. Tickets for the single lectures will be 1s. 6d. to members, and 2s. to non-members. For the cookery lessons, 10s. the course to members, and 12s. non-members. Due notice and syllabus of each lecture will be sent to members when the matter is settled, meanwhile any suggestions for classes and lectures, &c., will be received from members and considered with pleasure.

### Greek Ladies as Red Cross Nurses.

It may be in the remembrance of our readers that we published an article from an English nurse at Athens under this heading in The Hospital for July 10th, page 135 of The Hospital "Nursing Mirror." We hope that all who are interested in the questions dealt with will take the trouble to look up that article and to read it again carefully. At the end of August we received a letter from an Englishman holding an important official position in Athens, who took exception to the article written by the English nurse, but did not correct any statement in it, or point out any errors of fact. We, therefore, wrote to him in response to a further letter which he sent, stating we should be glad to publish a letter on the subject if he would confine it to a specific statement of his opinion of the nursing and nurses under the *Daily Chronicle*, and a categorical and definite denial of all or any points he might disagree with in the original article. We pointed out that we took special pains to satisfy ourselves of the *bona fides* of our correspondent, and the publication of the article was in our opinion justified by the confirmation of much that was said which has since reached us from reliable quarters. We added that we should be glad to publish anything that he might send in favour of the nurses who went out to Greece, but we considered that a letter to carry weight should be signed with his own name. In response to this letter the gentleman referred to writes under date of October 7th to the effect that had the nurse signed her own name it would have been more natural for him to sign his, and that, as it is, he would not have had the slightest objection to doing so, not being an incompetent judge as to the facts with which the original article dealt, having spent much of his time in the hospitals and amongst the nurses during their stay in Greece. He then goes on to say, "As, however, you are unwilling to publish the letter, and as I have no wish to be dragged into a controversy which cannot possibly do any good, it is with regret that I shall now be obliged to bring the letter to the notice of the authorities under whom the writer serves, for being in Government employment makes it the less excusable that she should have written such a letter. Her superiors must be the judges as to whether or not the letter is a proper one for her to have written under any circumstances whatever, more especially being employed in a Government hospital as she is." Our correspondent signs himself "A Knight of Justice of the Order of St. John of Jerusalem in England."

The facts being as stated, we now publish the original letter sent by the gentleman referred to, which is dated Athens, August 21st, 1897, and is as follows:—

"I have read with unmitigated surprise and regret the letter written by an 'English Nurse at Athens,' which appeared in the 'Nursing Mirror' of July 10th. I confess I do not understand the object such a letter can serve, emanating as it professes to do from a member of a society whose motto should be 'Peace and goodwill,' and whose duty certainly does not lie in the direction of finding fault with the shortcomings of countrywomen and colleagues, especially in a foreign land, even supposing that such shortcomings can be satisfactorily proved. The greater part of the letter, which throughout shows a surprising amount of ignorance of the subject of which it treats, is occupied with mere gossip, recapitulating what are generally known to be a nurse's ordinary duties under somewhat exceptional pressure, which a conscientious nurse is proud to perform without any flourish of trumpets. The remainder of the letter contains little else than self-glorification, and a sad propensity for running down her sisters' merits. The good work done by the English nurses sent out by her Royal Highness the Princess of Wales and by the committee of the *Daily Chronicle* (to neither of which bodies, I understand, the writer belonged) is acknowledged on all sides, and needs no champion; but it is regrettable to find that any so-called colleague who professes to have had her time fully occupied with her duties, and who, although placing a wide barrier between herself and her English sisters, yet professes to be an Englishwoman, should descend to such invidious comparisons and make such apparent insinuations as are contained in 'An English Nurse's' letter of July 10th. Her statements need no refutation, they speak for themselves, and appear to be equally in bad taste whether looked at from a Greek or from an English point of view."

We have to point out that it is a pure assumption on the part of our correspondent when he states that the writer is in Government employment, but, if she were, we have sufficient belief and confidence in the justice of the present Secretary of State for Foreign Affairs to believe that he would resent with righteous indignation any attempt made to bully or injure a defenceless Englishwoman faithfully discharging her duties in a foreign country. It will be seen, as we have pointed out to our correspondent, that although his letter just given contains various expressions of opinion, which, over a pseudonym, are of no importance, and can bear no weight, it in no way traverses any single statement made in the article to which it refers, namely, the one published in The Hospital "Nursing Mirror" for July 10th, 1897. Despite the request contained in our letter which we addressed to our correspondent, asking for a statement of any point in which the article in question was in error, he has failed to indicate any erroneous statement therein. In regard to his threat—for that seems the proper word—"to bring the letter to the notice of the authorities under whom the writer serves," we point out to our correspondent that he has not received and will not receive from us any intimation of the name of the writer of the article. If, however, he should have discovered from any other source the name of the writer, and in case he attempts to do her an injury by drawing her employers' attention to the article in question, we shall in turn draw attention in the proper quarter to the fact that the article referred to, as far as in one particular paragraph it criticised what he speaks of as "the shortcomings of the English nurses," did but give expression to a feeling which was widespread in Athens at the time, was the subject of common talk, and ought to have been perfectly well known to himself from the official position which he occupies there. We may add, as our readers will find on referring to the article, that it was a fair summary of the opinions held by the Greek ladies who cheerfully bore the greater portion of the burden of nursing their wounded fellow-countrymen during the late war, and that no impartial and reasonable person who reads it can take any exception to its language or to the sentiments which it expresses, always bearing in mind the fact that it represents the case from the point of view of the Greek ladies in question.

### Where to Go.

Special Exhibition of Nursing Appliances, 74, Regent Street, W.—Messrs. Southall Brothers and Barclay are holding an exhibition for nursing appliances from October 14th to 28th. It is open daily from ten a.m. to half-past six p.m. Miss Ede, the London representative of the firm, is in attendance of the hospital department, and tea is served from three to half-past six.

South-West London Polytechnic Institute, Monresa Road, Chelsea, S.W.—On October 9th Miss A. Wadmore, LL.A., began a course of 10 lectures on "Sanitation" at this institute. A lecture will be given weekly on Saturday evenings at seven p.m.

The quarterly meeting of the General Council of the Royal British Nurses' Association will be held at the office of the Corporation, 17, Old Cavendish Street, W., on Friday October 15th, 1897, at five p.m.

### The Book World for Women and Nurses.

[We invite Correspondence, Criticism, Enquiries, and Notes on Books likely to interest Women and Nurses. Address, Editor, THE HOSPITAL (Nurses' Book World), 28 & 29, Southampton Street, Strand, London, W.C.]

ELEMENTARY PHYSIOLOGY FOR NURSES. By C. F. MARSHALL, M.D., B.Sc., F.R.C.S. (London: The Scientific Press. 1897. Price 2s.)

There will no doubt always be a difference of opinion as to whether knowledge should be boiled down and presented in the form of an essence or taken raw. For people who have plenty of time, or for those who take up such a subject as physiology as a special study, a book of many words which presents the subjects with which it deals in many aspects is probably the best. But the modern nurse is not by any means a person with much spare time, nor can she afford to make a special study of physiology, for her special study must always be her work. Still something of physiology she must know. If she would take an intelligent interest in her daily work she must have some idea of the nature of the processes of life, and thus it happens that a short and pithy handbook such as Dr. Marshall has produced is likely to be of great value to her. He evidently understands the sort of knowledge that nurses want, and has concerned himself chiefly with producing a book which shall be readable and yet concise, simple and yet true. In this he has been very successful. The book is elementary in this sense, that it does not go into abstruse questions, and that it deals almost exclusively with what is known with certainty on the subject. But it is not a mere popular exposition, nor is it made elementary at the cost of making it imperfect, but rather by the care that has been taken to cut out all that is not essential. It seems to us to give what nurses want and but little else, and in doing that the author has well fulfilled the task he set before him.

FEVERS AND INFECTIOUS DISEASES: THEIR NURSING AND PRACTICAL MANAGEMENT. By WILLIAM HARDING, M.D.Edin., M.R.C.P.Lond. (London: The Scientific Press. 1897. Price 1s.)

This is a refreshing book, and comes as a relief to the weary reviewer, for it is evidently written out of fulness of knowledge, and concentrates in its eighty-five small pages the results of an immensity of experience. Moreover, it is written in English, and is written in such a way that it can be understood without having to run to a medical lexicon every few minutes. This is, perhaps, a small thing to some, but it is important to many who may use the book. For instance, after describing the symptoms of hæmorrhage in typhoid fever, the writer says, "The doctor should be at once communicated with. Cold should be applied to the abdomen over the right iliac region, that is, over the lowest part of the right side of the abdomen," &c. That is simple language which explains itself as it goes on. The book is divided into three parts. The first is on infection, the second on disinfectants, and the third, which occupies more than half the book, is on the nursing of infectious diseases. In the first part we are given a short sketch of the nature of infectious diseases, in which the necessity of good nursing and of the proper disinfection of all that has been in contact with the patient is insisted on. Many useful details are entered into in regard to the stages of the eruptive fevers, the duties of the nurse—to herself as well as to others—the sick-room, ventilation, temperature, &c. All the minor details of fever nursing are described, the giving of enemata, the application of cold in the form of packs, baths, compresses, and ice; of heat in the form of vapour baths and hot packs; and the management of various symptoms. The second part, on disinfectants, deals with their application to the patient and his discharges, the room, and the nurse, and finally with the treatment of the body after death. The third part is devoted to a very pithy and readable account of the various infectious diseases, giving under each not only a sketch of the symptoms and progress of the disease in question, but the points which have to be borne in mind in regard to the nursing. We can speak very highly of this little book. It is pleasant to read and is full of information. It is but a shilling's-worth, but it is worth far more than a shilling.

MARIE HILTON. By J. DEANE HILTON. (London: Isbister and Co., 1897.)

In "Marie Hilton: Her Life and Work" Mr. John Deane Hilton has, in the volume before us, executed what is always a difficult task, namely, describing the life work of a parent with judgment and with taste. It was in 1868 that the Hilton family settled in the East-end of London in Ratcliff, and this book is a record of the work which they did among the poor of that district. "To produce," says Mr. Hilton, "a realistic impression of Ratcliff in the sixties would require the Titanic strength of Zola." The dirt, the squalor, the swarming masses, the overcrowded dwellings, the naked children, the disease, the vice, crime, and misery which are still a reproach to our civilisation were far worse some thirty years ago. The visitor in those days had to endure discomforts and witness scenes hideous, abominable, not to be described. "What to do with a population plunged in this awful state of filth, depravity, and ignorance seemed an insoluble problem." The volume contains most painful stories, none, perhaps, more painful than that of the Harrow boy dead of exposure and dissipation, lying on the naked boards of a miserable garret, his head pillowed in his old school cap. Marie Hilton was overwhelmed with the terrible pathos of this wasted life. She thought of his early surroundings, of his happy schooldays, and the end, here, desolate and degraded. This social wreck had been brought about by drink, and in chapters ten and eleven we have Mr. Hilton's views on this question. When the Hiltons began, in the sixties, to preach abstinence to the working men, they frequently needed the protection of the police. Undaunted, they continued their work, visiting the poor, establishing missions, sewing classes, soup kitchens; and in February, 1891, the well-known Crêche in Stepney Causeway was opened. Into the vexed question of the Crêche system we have no space to enter. It is amazing and, after the lapse of years, astonishing to observe that when Mrs. Hilton's Crêche was first opened there were some who insisted that no unbaptized child should be admitted. "Let," says Mrs. Hilton, "the same restriction be applied to a hospital and its absurdity becomes apparent at once. Imagine, for instance, a patient with a fractured limb being refused admittance because his religious views were held to be unsound by the management." In spite of opposition Mrs. Hilton succeeded, for her system appealed to the deep instincts of human nature. Mr. Hilton quotes these words of Hesba Stretton to express the spirit in which his mother undertook the charge of the infants, "the fragile beginnings of a mighty end," which were brought to her door. The volume, which is published by Isbister and Co., we cordially recommend to the notice of our readers.

### BOOKS RECEIVED.

T. FISHER UNWIN.

"Masters of Medicine." Edited by Ernest Hart, D.C.L.
"John Hunter." By Stephen Paget.

WILLIAM CLOWES AND SONS.

"Sanitation and Health." By Brig.-General R. C. Hart., V.C., C.B. Revised by Brig. Surgeon-Lieut.-Colonel T. H. Hendley, C.I.E.

FLETCHER, RUSSELL, AND CO.

"The Commercial Uses of Coal Gas." By Thomas Fletcher, F.C.S.

### How Nurses may Help the Hospitals.

Nurses will be the first to recognise and admit the duty they owe to the hospitals in which they have obtained their training, and without which they could never have become efficient or trained nurses. We have had good cause to welcome the co-operation of trained nurses on more than one occasion in connection with movements in which they were interested, where their sympathies were aroused. We think, therefore, that a large number of our readers, and especially private nurses, may like to know something of an organisation just instituted in support of the Prince of Wales's Hospital Fund, with the object of securing without difficulty or risk the support of an army of small givers who can only spare an annual subscription of a few shillings a year each. The Prince of Wales's Hospital Stamps have had a large and encouraging sale, but many people have abstained from purchasing them because they have felt a difficulty as to how to keep them afterwards. To remove this objection, and to prevent the abuses which have often attached to attempts to raise money for hospitals from small subscribers, a subscription book and stamp album which can be carried in the pocket is about to be published, and will be ready on October 23rd.

The object of the subscription book and stamp album is to enable subscribers of small amounts from 1s. to 10s. per annum to possess evidence of the fact that they are regular subscribers to the Prince of Wales's Hospital Fund and the metropolitan hospitals. It will contain a portrait of the Queen and the Prince of Wales; a certificate from Lord Rothschild, the treasurer of the Fund; the approval of the Queen, and the views of the Prince of Wales and the Duke of York. Each subscription book is of a size to contain twenty years' subscriptions of 1s. or 2s. 6d. per annum, or ten annual subscriptions of 5s., or five of 10s. per annum. These subscriptions are payable by the purchase of hospital stamps, which are then affixed to the pages assigned to them in the subscription book, the year for which each subscription is given being stated at the right-hand top corner of each stamp. There must be a large number of nurses who would like to help the hospitals by making these subscription books known to members of the public with whom they may come in contact, and who would not otherwise be likely to hear of this excellent plan, by which a large annual income may readily accrue to the London hospitals. Of course these subscription books and stamp albums with the stamps, which will be on sale by chemists, booksellers, stationers, newsagents, and stamp sellers, will be supplied to the public through these channels under a business arrangement made with the publishers. It would be unreasonable and undesirable that those trained nurses who may wish to take a part in this movement should be placed in a less favourable position than the trade should they voluntarily undertake to do their best to make the movement a success. It is therefore proposed that a similar business arrangement shall be effected between the nurses and the publishers. It has been suggested that sisters and nurses who have some leisure and influence may be willing to offer their services as centres of communication through which nurses may be approached and the whole movement set agoing. A small committee might perhaps be constituted with this object.

We have much pleasure in bringing the subject to the notice of our readers, and should be glad if any who may be willing to co-operate will send their name and address, the institution to which they are or have been attached, together with any suggestions or criticisms which they may desire to offer, to the Editor, at 29, Southampton Street, Strand, W.C. We should like to receive these replies by Monday, the 18th inst., so that fuller details may be published and if possible, a plan organised in time for publication in next week's issue of The Hospital. The assistance of only a certain number of nurses can be accepted, and it would seem to be the fairest course to give precedence to applicants in the order in which their letters may be received at The Hospital office. All envelopes should be marked "Stamp Album" in the left-hand corner at the bottom.

### Everybody's Opinion.

[Correspondence on all subjects is invited, but we cannot in any way be responsible for the opinions expressed by our correspondents. No communication can be entertained if the name and address of the correspondent is not given, or unless one side of the paper only is written on.]

### UNSEEMLY AMUSEMENTS.

"A Lady Superintendent" writes: As a large ball is about to be given in a certain infirmary, on which occasion each nurse is to be allowed to invite a gentleman, whose name is to be submitted to the committee, it is high time that a protest should be made against the frivolous tone that seems to be steadily increasing in the nursing profession, so that this work is taken up by many as a lively and gay method of passing time instead of the noble and sacred work of caring for the diseased and disabled bodies of our fellow human beings. Is it commonly respectful to the sick and dying that doctors and nurses, the representatives of what ought to be the most noble professions, should be engaged in dancing the evening out in a building set apart for the sick and under the same roof with pain and illness, where perhaps several may be breathing their last, nay, even dead bodies being carried out. Surely dancing and death are not "compatible," and if one could put themselves in the place of those who may be watching by the bed of some near relation on the "dangerous list," what would be one's own feelings on such an occasion to hear the sound of music and dancing, or even to know that such were going on under the same roof. There is another aspect from which this may be viewed. Is it not seriously detrimental to discipline and hospital etiquette that nurses who have to work under doctors in any institution should be so familiar as to dance with them, more especially when they may have to work together for years? Though nurses may be acquainted with doctors outside, and in many cases rank higher in the social scale, that does not alter their relations in work. The doctors in many cases are more to blame than the nurses, though any woman who seriously takes up nursing with the right motives will have more self-respect than to allow any unbusinesslike gossip and familiarity to take place between herself and the doctors for whom she works; but if doctors, too, would remember their position it would make it easier for the nurses.

### Minor Appointments.

Cockermouth.—Miss Margaret B. Robinson was appointed Queen's Nurse for Cockermouth on September 28th. She was trained at Salisbury Infirmary, and for the last four years has been on the staff of the North-East Manchester District Nurses' Home.

Rochford Union Workhouse Infirmary, Essex.—The Charge Nurse appointed to this infirmary on October 5th was Miss Harriett Carswell. She has trained at Mill Road Infirmary, Liverpool, and has been head nurse at Alcester Union Infirmary, Warwickshire.

Clatterbridge, near Birkenhead.—On October 6th Nurse Charlotte H. Smith, trained at Eston Sanatorium, York, was appointed Staff Nurse of the Isolation Hospital. She had previously been assistant nurse at City Hospital, Priory Road, Liverpool.

### For Reading to the Sick.

"HOLY ZEAL."

"LET this mind be in you which was also in Christ Jesus." —*John* ii. 17.

**Verses.**

In a service which Thy will appoints
There are no bonds for me;
For my inmost heart is taught the truth
That makes Thy children free;
And a life of self-renouncing love
Is a life of liberty. —*A. L. Waring.*

And now we fight the battle,
But then shall wear the crown
Of full and everlasting
And passionless renown;
And now we watch and struggle,
And now we live in hope,
And Sion in her anguish
With Babylon must cope;
But He whom now we trust in
Shall then be seen and known,
And they that know and see Him
Shall have Him for their own.
—*Bernard of Cluny.*

Onward, therefore, pilgrim brothers!
Onward with the Cross, our aid!
Bear its shame, and fight its battle,
Till we rest beneath its shade.
Soon shall come the great awaking,
Soon the rending of the tomb;
And the scattering of all shadows,
And the end of toil and gloom.
—*S. Baring-Gould.*

**Reading.**

Zeal is a principle; enthusiasm is a feeling. The one is the spark of a sanguine temperament and over-heated imagination. The other, a sacred flame, kindled at God's altar and burning in God's shrine.—*Vaughan.*

Such was the holy, heavenly zeal of our Great Exemplar. His were no transient outbursts of ardour, which time cooled and difficulties impeded. His life was one indignant protest against sin; one ceaseless current of undying love for souls, which all the malignity of foes and unkindness of friends could not for one moment divert from its course. Even when He rises from the dead, and we imagine His work is at an end, His zeal only medidates fresh deeds of love. "Still His heart and His care," says Goodwin, "is upon doing more. Having now despatched that great work on earth, He sends His disciples word that He is hastening to heaven as fast as He can to do another." While zeal is commendable, remember the Apostle's qualification, "It is good to be zealously affected always in a good thing." There is in these days much base coin current, *called* "Zeal," which bears not the image and superscription of Jesus. There is zeal for church membership; zeal for party creeds and dogmas; zeal for figments and non-essentials. "From such turn aside." Your Lord stamped with His example and approval no such counterfeits. His zeal was ever brought to bear on two objects, and two objects alone—the glory of God and the good of men. Be it so with you. . . . . Have a zeal for others. Dying myriads are around you. As a member of the Christian priesthood, it becomes you to rush in with your censer and incense between the living and the dead, that the plague may be stayed.—*By the Author of the "Night and Morning Watches."*

### Notes and Queries.

**The contents of the Editor's Letter-box have now reached such unwieldy proportions that it has become necessary to establish a hard and fast rule regarding Answers to Correspondents. In future, all questions requiring replies will continue to be answered in this column without any fee. If an answer is required by letter, a fee of half-a-crown must be enclosed with the note containing the enquiry. We are always pleased to help our numerous correspondents to the fullest extent, and we can trust them to sympathise in the overwhelming amount of writing which makes the new rules a necessity. Every communication must be accompanied by the writer's name and address, otherwise it will receive no attention.**

*Poisoning.*

(21) I wish to ask you if Dr. Murrell's pocket-pook, entitled "What to do in Cases of Poisoning," is suitable for a nurse; (2) and what the cost would be.—*Nurse.*

(1) Dr. Murrell's book is a standard work, and most suitable for a nurse. (2) The Scientific Press, 28 and 29, Southampton Street, Strand, will send a copy post free for 3s. 6d.

*Address of Apothecaries' Hall.*

(22) Will you kindly let me know the address of the Apothecaries' Hall? (2) Also the most suitable book for a nurse to study dispensing from?—*Nemo.*

(1) The address is Blackfriars, E.C. (2) Dispensing cannot be learned from books, but a series of valuable articles on this subject have recently appeared in THE HOSPITAL. The numbers containing it can be obtained on application to the manager.

*Training versus Work.*

(23) Can you inform me if a young lady, thoroughly healthy and strong, could obtain few months' nursing experience in a good hospital, sufficient to fit her for a post of under matron in a high class boys' school, in return for her services?—*Labore et Honore.*

The only way to obtain a few months' training in a good hospital is to become a paying probationer. You could learn comparatively nothing of medical nursing in a few months, but might acquire a knowledge of ward arrangements and of surgical dressings, and this might be an advantage in applying for the post.

*Aged Monthly Nurse.*

(24) A monthly nurse, aged 64, who has been nursing for 38 years, is now suffering from rheumatism, and would be glad to hear of any society which would afford her permanent help. She is not likely to be able to work much again, and has no private means and no relatives who could help.—*H. D.*

See Homes and Charities in Burdett's "Hospitals and Charities" for 1897, p. 840.

*Paying Mental Case.*

(25) Will you kindly inform me if there is an asylum where a light mental case could be taken on payment of a small weekly sum?—*Anxious.*

Paying patients are frequently taken in the county and borough asylums when there is room, the terms varying from 14s. to £1 1s. per week, according to the arrangements made with the medical superintendent. This, perhaps, removes your objections to a public asylum. Write in any case to the medical superintendent of your county, and he will probably be able to give you the information you want, even if this arrangement is not suitable.

*Training as a Male Nurse.*

(26) I should be much obliged to you if you would give me your advice as to obtaining training as a male nurse. I am willing to pay for instruction and practice, as my object is to get on rapidly; but whatever you may suggest will be thankfully received. I have attended the St. John's courses, and am what people probably would call well educated. I mention this so that you may advise me accordingly. You will be giving me very real help if you can indicate a way, as I find much difficulty in my search for good practice and tuition.—*G. P. Kling.*

There is, unfortunately, no general hospital in England that trains male nurses. The National Hospital for the Paralysed and Epileptic, Queen's Square, Bloomsbury, take a few probationers, and possibly the Hamilton Association for Supplying Male Nurses, 57, Park Street, Grosvenor Square, W., might give you some help.

*Incurable Children.*

(27) Will you kindly tell me (1) of any institution which would receive an incurably crippled girl of 13 years old; and (2) whether any children's hospital would take a girl of 14 suffering from curvature of the spine? If not, where would it be best to send her? The parents of both girls are very poor.—*M. C.*

(1 and 2) See list of homes for incurables, p. 906, also list of orphanages, homes, and charities, p. 840, in Burdett's "Hospitals and Charities" for 1897 (at this office). Most probably you will be able to find one amongst the number to help.

*Probationer's Entrance Examination.*

(28) Would you kindly tell me what examinations I should have to pass before entering as probationer for sick nursing in hospital or infirmary?—*E. B.*

The examination is a very simple one in elementary subjects—that is, reading, writing, &c.

*Sanitary Inspection.*

(29) 1. Will you kindly tell me the requirements—classes, certificates, &c.—for post of sanitary inspector? 2. Is the salary the same for a woman as for a man, and about what is the salary?—*Annie Scott.*

1. The Secretary of the Sanitary Institute, Margaret Street, W., or of Bedford College, Baker Street, will give you full particulars. 2. The salary will, of course, depend on circumstances and locality. A lady inspector recently appointed in London receives a salary of £105 per annum.

## Figures and Tables

**Figure f1:**
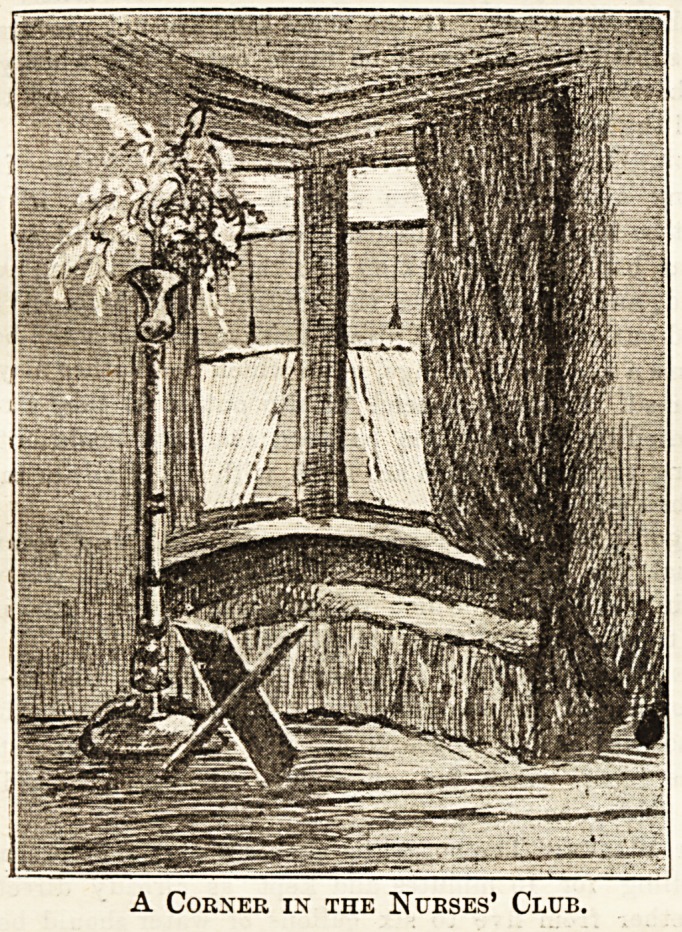


**Figure f2:**